# Prognostic significance of downregulated expression of the candidate tumour suppressor gene *SASH1* in colon cancer

**DOI:** 10.1038/sj.bjc.6603452

**Published:** 2006-10-31

**Authors:** C Rimkus, M Martini, J Friederichs, R Rosenberg, D Doll, J R Siewert, B Holzmann, K P Janssen

**Affiliations:** 1 Department of Surgery, Klinikum rechts der Isar, Technische Universität München, Ismaninger Str. 22, Munich81675, Germany

**Keywords:** colorectal cancer, prognosis, signalling adapter protein, metastasis

## Abstract

The gene *SASH1* (SAM- and SH3-domain containing 1) has originally been identified as a candidate tumour suppressor gene in breast cancer. *SASH1* is a member of the *SH3-domain containing expressed in lymphocytes* (*SLY1*) gene family that encodes signal adapter proteins composed of several protein–protein interaction domains. The other members of this family are expressed mainly in haematopoietic cells, whereas *SASH1* shows ubiquitous expression. We have used quantitative real-time PCR to investigate the expression of *SASH1* in tissue samples from 113 patients with colon carcinoma, and compared the expression with 15 normal colon tissue samples. Moreover, nine benign adenomas and 10 liver metastases were analysed. Expression levels of *SASH1* were strongly and significantly reduced in colon cancer of UICC stage II, III, and IV, as well as in liver metastases. Moreover, SASH1 was also found to be downregulated on protein levels by immunoblot analysis. However, *SASH1* expression was not significantly deregulated in precancerous adenomas and in earlier stage lesions (UICC I). Overall, 48 out of 113 primary colon tumours showed *SASH1* expression that was at least 10-fold lower than the levels found in normal colon tissue. Downregulation of *SASH1* expression was correlated with the formation of metachronous distant metastasis, and multivariate analysis identified SASH1 downregulation as an independent negative prognostic parameter for patient survival. This study demonstrates for the first time that expression of a member of the *SLY1*-gene family has prognostic significance in human cancer.

Many of the molecular changes associated with tumour formation have been defined for colorectal cancer, owing to the high frequency of the disease and the relatively simple access to virtually all tumour stages by endoscopy (Fearon and Vogelstein, 1990; Kinzler and Vogelstein, 1996). However, many more genes, some still uncharacterised, are likely to be involved in tumour progression and metastasis formation. We have focused on the gene *SASH1* (*SAM- and SH3-domain containing 1*) that has previously been described as candidate tumour suppressor gene in breast cancer (Zeller *et al*, 2003). *SASH1* was mapped to chromosome 6q24.3, loss of heterozygosity (LOH) of this region (occurring in 30% of primary breast cancers) was associated with poor survival and increase in tumour size. Moreover, a strong reduction of *SASH1* expression was observed in the majority of breast tumours when compared to normal mammary epithelia (Zeller *et al*, 2003). Loss of chromosomal region 6q24–25 has also been detected in a study on primary colorectal tumours, and was specifically associated with invasive tumours extending to the serosal fat (Alcock *et al*, 2003). However, the predictive capacity of *SASH1* deregulation in human cancer has not been determined previously. The domain structure and strong sequence similarities places *SASH1* in the *SH3-domain containing expressed in lymphocytes* (*SLY1*) family of signal adapter proteins, which was first identified in haematopoietic cells (Beer *et al*, 2001), and comprises in addition the proteins SLY1, and HACS1 (or SLY2, NASH1). SLY1 is involved in regulation of the adaptive immune system (Beer *et al*, 2001, 2005). HACS1 (or NASH1) is expressed in mast cells, and was reported to be differentially expressed in malignant haematopoietic cells (Claudio *et al*, 2001; Uchida *et al*, 2001; Zhu *et al*, 2004). Whereas the expression of SLY1 and HACS1 seems to be restricted to haematopoietic cells, *SASH1* shows ubiquitous expression in human tissue (Zeller *et al*, 2003). Here, we provide the first evidence that downregulation of *SASH1* constitutes an independent prognostic parameter in colon cancer.

## PATIENTS, MATERIALS AND METHODS

### Patients

Informed, written consent regarding the use of the tissue samples was obtained from each subject before the study. Tissue samples were obtained from 113 patients admitted to our Department of Surgery with the diagnosis colon carcinoma. The group consisted of 69 male and 44 female patients, mean age was 64 years. None of the patients suffered of a known second neoplastic disease; only complete resected tumours (R0) were included in the study. Median survival after surgery was 91 months (range: 44–131 months). During this period, 38 patients died owing to tumour-related causes. Disease recurred in 15 patients, 34 patients developed metachronous distant metastases, and 23 patients showed disease progression. Tumour localisation was: ascending colon (41 cases), transverse colon (12 cases), descending colon (18 cases) and sigmoid colon (42 cases). Tumour grading was: G1 (three cases), G2 (74 cases), G3 (33 cases) and G4 (three cases). Tumour stages according UICC classification were: stage I (12 cases), stage II (45 cases), stage III (23 cases), and stage IV (33 cases). Sixty-five patients had no adjuvant treatment, and 48 patients received systemic chemotherapy. As a control, we examined normal colon tissue (15 patients), benign colonic adenomas (nine patients), and liver metastases from 10 patients. Samples were frozen in liquid nitrogen immediately after surgery and stored at −80°C.

### RNA Isolation from cell lines and tissue samples

To establish and validate the quantification of *SASH1* expression the following human cell lines were used: HEK293 (embryonic kidney epithelial cells), HeLa (cervical carcinoma), SKOV-3 (ovarian adenocarcinoma), CaCo2 (grade II colorectal adenocarcinoma), HT29 (grade I colorectal adenocarcinoma), Jurkat (T-lymphocyte from acute T-cell leukaemia), and Ramos (B-lymphocyte from Burkitt's lymphoma). For RNA isolation from tissue, we used 40 sections of 12 *μ*m thickness (surface approximately, 1–2 cm^2^). Immunochemistry (haematoxylin (H) and eosin (E) staining) was performed on each first and last section to ensure tumour content above 70%. Accordingly, frozen sections from resected normal colon tissue (as certified by an experienced pathologist) were subjected to the same protocol. RNA was isolated using the RNeasy Kit (Qiagen, Hilden, Germany), quantified, and checked for degradation on a denaturing formaldehyde–agarose gel. cDNA preparation was performed according to standard procedures, using SuperscriptII H-Reverse Transcriptase (Invitrogen, Karlsruhe, Germany) and oligo-dT primers (pd(N)6; Roche Alameda, CA, USA).

### Quantitative real-time PCR

Expression of *SASH1* and *SLY1* transcripts was determined by real-time reverse transcriptase–polymerase chain reaction (RT—PCR) using the ABI PRISM 7300 sequence detection system (Applied Biosystems, Foster City, CA, USA) with the dye SYBRGreen I. Expression of the housekeeping gene *HPRT* was used as internal reference.

• HPRT-F:  5′-GCT TTC CTT GGT CAG GCA GTA TAA T-3′
• HPRT-R:  5′-AAG GGC ATA TCC TAC AAC AAA CTT G-3′
• SASH1-F:  5′- CGG GAA AGC GTC AAG TCG GA-3′
• SASH1-R:  5′- ATC TCC TTT CTT GAG CTT GAG-3′
• SLY1-F:  5′- TCC AGC AGC TTC AAG GAT TT-3′
• SLY1-R:  5′- CAT CTT GCC CAT CTT CCT GT-3′

### Statistical analysis

Analyses were performed using SPSS version 9.0 (SPSS, Munich, Germany). Statistical significance was defined as *P*⩽0.05. As data sets were too small to assume normal distribution, nonparametric tests were used. The results were analysed with the Kruskal–Wallis test or Mann–Whitney *U*-test to check for significant differences between individual groups. Expression of *SASH1* was assessed in terms of survival by the Cox proportional hazards model using univariate and multivariate analysis. Significance was tested by *χ*^*2*^ analysis.

### Preparation of protein lysates

Resected tumours and normal colon tissue samples (as certified by an experienced pathologist) were snap-frozen in liquid nitrogen in lactate buffered Ringer's solution, and stored at −80°C. Tissue samples were thawn quickly and homogenised in pre-cooled Dounce homogenisers in TBST buffer (20 mM Tris/HCl, pH 8.5, 150 mM NaCl, 1% Triton X-100). After homogenisation, samples were extracted overnight at 4°C, and subsequently centrifuged for 1 h, 4°C, at 100 000 *g* in an ultracentrifuge (Beckman, Krefeld, Germany). Supernatants were collected, frozen in liquid nitrogen, and stored at −80°C. Protein concentration was determined with a Bradford Assay (Biorad, Munich, Germany).

### Immunoblotting and antibodies

Equal amounts (40 *μ*g) of protein lysates were boiled in Laemmli buffer for 5 min, separated on 10% polyacrylamide gels, and subjected to immunoblotting according to standard procedures (Towbin *et al*, 1979). Mouse monoclonal anti-*β*-tubulin antibody (Oncogene Research Products, Darmstadt, Germany) was used as loading control. A polyclonal rabbit anti-serum was generated by immunisation with two specific peptides corresponding to amino-terminal sequences of murine *SASH1*, and the serum was further affinity-purified with the peptides. A detailed description of the antiserum will be subject of a future publication. Briefly, the antiserum was used at 1 : 2000 dilution on the immunoblots (at 4°C, overnight), recognising a specific band of an apparent molecular weight of 180 kDa in normal human colon lysates. Secondary antibodies were horseradish peroxidase-conjugated goat anti-mouse IgG and goat anti-rabbit IgG (Jackson Immunoresearch, West Grove, PA, USA), visualised with an enhanced chemiluminescence substrate detection kit (Pierce, Rockford, IL, USA).

## RESULTS

**Figure 1:**
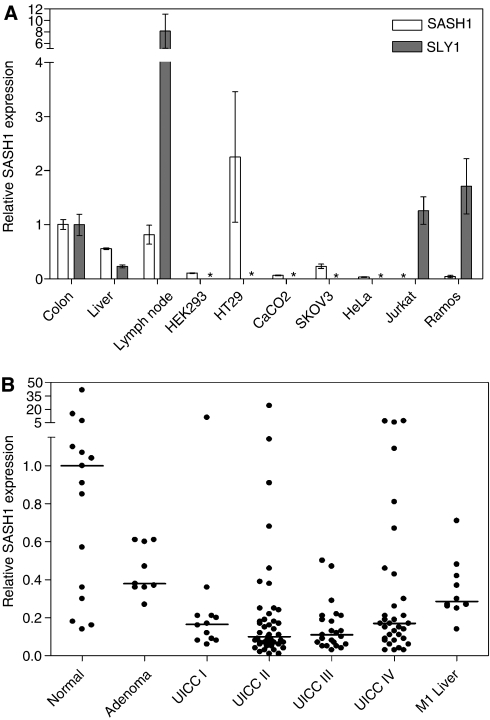
(**A**) Relative expression of *SASH1* and *SLY1* determined by qRT–PCR in normal human tissue (*n*=3 specimen) and cell lines, normalised to the expression in colon. Means±s.d. for three independent experiments. Asterisk: no expression detectable. (**B**) Relative *SASH1* expression in normal colon (*n*=15), benign adenomas (*n*=9), and primary colon tumours from stages UICC stage I (*n*=12), stage II (*n*=45), stage III (*n*=23), and stage IV (*n*=33). Moreover, liver metastases (*n*=10) and normal liver tissue (*n*=9) not shown was analysed. SASH1 expression was significantly different from normal tissue in: UICC stage II (*P*=0.031), UICC stage III (*P*=0.047), UICC stage IV (*P*=0.043), and in liver metastases (*P*=0.030). The bar indicates median expression for each group. Expression was normalised to the median expression of all normal colon samples.

We have first tested normal human tissues and cell lines by qRT–PCR for the expression of *SASH1* and its homolog SLY1. *SASH1* expression was detected in colon, liver and peripheral lymph nodes, and in reduced amounts as compared to normal colon in various cell lines of epithelial origin (HEK293, SKOV-3, CaCo_2_) (Figure 1A). Interestingly, expression in HT29 cells (corresponding to well-differentiated colorectal adenocarcinoma) was two-fold higher than in normal colon. *SASH1* expression was very low to undetectable in lymphoblastoid cells (Jurkat and Ramos cells) and HeLa cells. The expression patterns of *SASH1* and its homologue SLY1 were found to be mutually exclusive, as SLY1 was expressed in the lymphoblastoid cells, but undetectable in epithelial cells. Correspondingly, SLY1 showed highest expression in lymph nodes, and about 10-fold lower expression in colon. It has been reported that protein kinase C (PKC) is a regulator of *SASH1* expression (Lindvall *et al*, 2005). However, we could not observe any difference in expression of *SASH1* upon stimulation of PKC with phorbol 12-myristate 13-acetate (PMA)/ionomycin in all cell lines tested (not shown).

Next, we analysed primary colon carcinomas from 113 patients and compared the expression with 15 normal colon samples (Figure 1). In addition, nine precancerous lesions (adenoma) were analysed. *SASH1* was significantly downregulated in tumour stages UICC II, III and IV, but not in adenomas or UICC stage I cancers (Figure 1B). Forty-two per cent of all carcinomas (48 out of 113 samples) showed *SASH1* expression that was 10-fold lower in comparison to normal colon tissue. Of note, we detected high levels of expression (>5-fold of the median expression in normal colon) in five tumour samples (4%), and in three normal colon tissues (23%). However, *SASH*1 expression was not significantly correlated to the anatomical tumour site.

**Figure 2:**
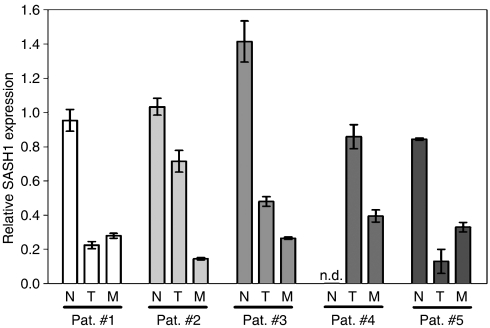
Relative expression of *SASH1* in matched normal tissue, primary colon tumours and liver metastasis from five patients, normalised to the median expression of all normal colon samples. Mean±s.d. is indicated for three replicate measurements. Note: Normal colon was not available for patient #4.

We then compared matched normal colon tissue, primary tumour and colorectal liver metastasis from five individual patients (Figure 2). In all cases, the expression of *SASH1* was significantly reduced in the liver metastasis as compared to normal colon tissue. In three cases, *SASH1* was already downregulated in the primary tumour, and in two patients, the *SASH1* mRNA level of the liver metastasis was significantly lower than that of the matched primary tumour (*P*<0.01). As the expression levels of *SASH*1 in normal human liver were not well established so far, we have compared liver metastases to normal liver tissue from nine patients. We could confirm that *SASH1* was significantly downregulated in the metastatic tissue also in comparison to the normal surrounding liver tissue.

**Figure 3:**
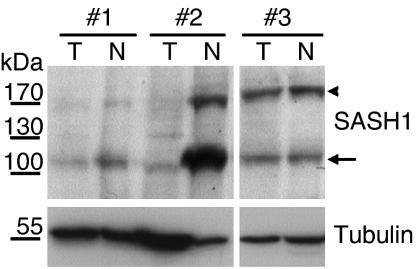
SASH1 protein expression in tumour (marked ‘T’) and matched normal tissue (marked ‘N’) from three representative patients. The SASH1 signal has an apparent molecular weight of 180 kDa (arrowhead). *SASH1* expression was lower in tumour than in corresponding normal tissue in eight out of 10 tested patients, as seen in patients #1 and #2. In case #3, *SASH1* expression was unchanged in the tumour as compared to normal colon. Note the presence of a prominent additional band with higher electrophoretic mobility, which is also reduced in tumours (arrow). Tubulin staining as loading control indicated in the lower panel.

The observed downregulation of *SASH1* expression may not necessarily be found on protein levels. Therefore, we have generated a polyclonal rabbit anti-serum that specifically recognises a peptide epitope of SASH1. In a restricted subset of patient samples (*n*=10), where sufficient material (>50 mg) from both normal and tumour tissue from the same patient was available for protein extraction, we have tested expression by immunoblotting. *SASH*1 signals were visible at 180 kDa (Figure 3, arrowhead), somewhat exceeding the predicted molecular weight of 140 kDa. Downregulation of SASH1 protein was detectable in eight out of 10 tested tumours. Figure 3 shows representative examples: tumours from patients #1 and #2 show strong downregulation of SASH1, whereas tumour #3 has an equal expression of SASH1 in normal and cancer tissue. Interestingly, a prominent second band with higher electrophoretic mobility (about 110 kDa) was detected in tumour lysates with the anti-SASH1 anti-serum, especially strong in normal tissue from patient #2 (Figure 3, arrow). This lower band may correspond to a degradation product, or to a splice variant of SASH1. After preincubation of the immunoblot with a 10-fold molar excess of the peptides against which the antiserum was generated, both bands were no longer visible, demonstrating the specificity of the immunoreactive bands (not shown).

SASH1 transcript expression was not correlated with disease recurrence, but there was a significant correlation of SASH1 expression with metastasis formation: SASH1 downregulation was correlated with the development of metachronous metastasis, defined as metastases occurring more than 6 months after resection of the primary tumour (*P*=0.014, Mann–Whitney *U*-test). SASH1 downregulation was also associated with the progression of synchronous metastasis (*P*=0.046, Kruskal–Wallis *H*-test). The group of 113 patients was then statistically evaluated by multivariate regression analysis for overall survival using the following factors: age, sex, disease recurrence, pT and nodal status (pN) status, tumour grading, and *SASH1* expression (Table 1). *SASH1* expression below cutoff (10-fold lower than in normal colon) independently predicted survival in this multivariate analysis using the Cox proportional hazard model, with a relative risk of 2.9 (*P*=0.005). This analysis also retained known independent predictors for survival, such as disease recurrence, pN, and tumour grading, whereas age and sex were not retained. The pT status was retained in univariate, but not in multivariate analysis as independent parameter.

## DISCUSSION

A tremendous amount of information has been achieved regarding the molecular and cellular changes associated with colon cancer. However, the overall survival rate for this disease has not substantially improved over the last 20 years (Parker *et al*, 1997), and patient prognosis still relies mainly on the penetration depth of the tumour and the spreading of the disease to other organs. The identification of new genes functionally involved in tumour development and progression may help to find alternative approaches for prognostic and diagnostic evaluation. Our study focuses on *SASH1*, a new candidate tumour suppressor gene of the *SLY1* family of signal adapter proteins. We have observed broad expression of *SASH1* in various normal tissues and epithelial cell lines, with the exception of lymphoblastoid cells. The observed expression pattern of *SASH1* was complementary to that of its homologue *SLY1*.

Interestingly, *SASH1* expression was significantly downregulated in invasive and metastasised tumours. The downregulation of *SASH1* expression was validated and reproduced independently by immunoblot analysis. *SASH1* expression was not reduced in adenomas and locally restricted, low-stage tumours (UICC I). Therefore, it is tempting to speculate that *SASH1* does not play a role in the early phases of tumour initiation, but is rather related to processes such as tumour invasion in the surrounding tissue, and dissemination of tumour cells to distant organs. Indeed, *SASH1* expression was clearly downregulated in liver metastasis, and the expression levels in the metastases were equal to or lower than the expression in the primary tumour from the same patient. Statistical analysis revealed that downregulation of SASH1 in the primary tumour correlated with the formation of metachronous, postoperative metastasis, and with the progression of synchronous metastases. Moreover, *SASH1* downregulation was retained as an independent negative prognostic factor for patient survival by multivariate analysis.

Currently, the molecular mechanisms that regulate SASH1 expression are unknown. Stimulation of PKC *in vitro* was reported to reduce *SASH1* expression in primary B-lymphocytes (Lindvall *et al*, 2005). Interestingly, PKC may play a role in colorectal cancer formation (Zhang *et al*, 2004), and the homologue SLY1 is regulated by PKC (Beer *et al*, 2005). However, we did not detect any regulation of SASH1 expression by PMA/ionomycin stimulation *in vitro* in various cell lines (HeLa, HEK293, HT29, Ramos). Alternatively, the downregulation of SASH1 in tumours may be explained by genetic mechanisms such as LOH or point mutations. Indeed, frequent LOH at the SASH1 genomic locus 6q24–25 has been reported in primary breast cancer, and also in one study in primary colorectal tumours, with specific association with tumour invasion to the serosal fat (Alcock *et al*, 2003; Zeller *et al*, 2003). However, despite extensive sequencing efforts, Zeller and co-workers have not been able to demonstrate invalidating mutations in the second allele that would explain the lack of expression of *SASH1* in breast cancer (Zeller *et al*, 2003). Thus, epigenetic mechanisms such as promoter hypermethylation may underlie the observed downregulation of *SASH1* expression.

In the patient collective, a subgroup of tumours displayed normal or elevated levels of *SASH1*. This might be explained by the different routes that lead to colorectal cancer. Sporadic cancers of the colon are characterised by different forms of genetic instability, which can manifest themselves in either chromosomal instability, or, less frequently, in microsatellite instability (MSI). Interestingly, *SASH1* expression was normal in a tumour from a patient diagnosed with hereditary nonpolyposis colorectal cancer (HNPCC) syndrome and MSI-high status (not shown). Even though sporadic MSI-high colon cancer differs from the rare hereditary forms (Jass, 2004), it is tempting to speculate that there may be a connection between normal SASH1 expression in tumours and high MSI levels. However, the tumour samples in this study were not generally characterised for their MSI levels. Therefore, further investigations are necessary to clarify this hypothetical correlation between MSI status and the expression of the tumour suppressor SASH1.

The functional consequences of downregulated *SASH1* expression are still unclear. The *SASH1* gene encodes a large protein of 1230 amino acids that contains two SAM (sterile α-module) domains, and one SH3-domain (*Src* homology domain 3). SH3 domains bind to proline-rich motifs in proteins (Pawson, 1995), SAM domains are able to self-associate or to interact with other protein domains (Kim *et al*, 2001, 2002). Both domains are frequently found in signal adapter proteins or scaffolding factors. Thus, the function of SASH1 may lie in the formation of multiprotein signalling complexes that regulate cell survival, proliferation, or migration. In conclusion, we have demonstrated that downregulation of *SASH1* expression in colon cancer is associated with metastasis and bad prognosis.

## Figures and Tables

**Table 1:** Multivariate Cox regression analysis of prognostic variables

**Variable**	**Relative risk**	***P*-value**
pT	—	NS
pN	2.124	0.001
Recurrence	3.735	<0.001
*SASH1* expression below cutoff	2.908	0.005
Grading	2.413	0.002

pN=nodal status; pT=primary tumour stage.
